# Evolution of Liquid Biopsies for Detecting Pancreatic Cancer

**DOI:** 10.3390/cancers16193335

**Published:** 2024-09-29

**Authors:** Ryan Munnings, Peter Gibbs, Belinda Lee

**Affiliations:** 1 Walter & Eliza Hall Institute of Medical Research, Parkville, VIC 3052, Australia; 2 Department of Medical Biology, University of Melbourne, Parkville, VIC 3052, Australia; 3 Department of Medical Education, Melbourne Medical School, Parkville, VIC 3052, Australia; 4 Western Health, Footscray, VIC 3011, Australia; 5 Peter MacCallum Cancer Centre, Parkville, VIC 3052, Australia; 6 Northern Health, Epping, VIC 3076, Australia

**Keywords:** pancreatic ductal adenocarcinoma, liquid biopsy, circulating tumour DNA, circulating tumour cell, circulating tumour exosome, proteomics, cancer screening

## Abstract

**Simple Summary:**

Pancreatic ductal adenocarcinoma (PDAC) is a highly lethal malignancy characterised by late diagnosis and poor prognosis, with current diagnostic and prognostic strategies proving insufficient. Liquid biopsy techniques, including circulating tumor DNA (ctDNA), circulating tumor cells (CTCs), exosomes, and proteomics, present potential avenues for enhancing PDAC diagnosis, prognosis, and management. A review of literature from 2019 to 2024 identified 49 relevant studies focusing on these biomarkers. Investigations into ctDNA, particularly in detecting mutant *KRAS*, have shown some diagnostic and prognostic potential, though its efficacy in early-stage disease remains limited. CTCs, whilst highly specific, exhibit similarly restricted diagnostic utility in early disease, and studies have yielded inconsistent prognostic findings. Exosomal research identifies diverse biomarkers with promising diagnostic and prognostic value. Importantly, proteomics demonstrates superior accuracy in PDAC diagnosis, including early-stage detection, and significant prognostic capacity, especially when combined with CA19-9 analysis. This review highlights the significance of continued research into liquid biopsy techniques to improve PDAC management and outcomes.

**Abstract:**

Pancreatic ductal adenocarcinoma (PDAC) is a lethal malignancy characterised by late diagnosis and poor prognosis. Despite advancements, current diagnostic and prognostic strategies remain limited. Liquid biopsy techniques, including circulating tumour DNA (ctDNA), circulating tumour cells (CTCs), circulating tumour exosomes, and proteomics, offer potential solutions to improve PDAC diagnosis, prognostication, and management. A systematic search of Ovid MEDLINE identified studies published between 2019 and 2024, focusing on liquid biopsy biomarkers for PDAC. A total of 49 articles were included. ctDNA research shows some promise in diagnosing and prognosticating PDAC, especially through detecting mutant *KRAS* in minimal residual disease assays. CTC analyses had low sensitivity for early-stage PDAC and inconsistent prognostic results across subpopulations. Exosomal studies revealed diverse biomarkers with some diagnostic and prognostic potential. Proteomics, although relatively novel, has demonstrated superior accuracy in PDAC diagnosis, including early detection, and notable prognostic capacity. Proteomics combined with CA19-9 analysis has shown the most promising results to date. An update on multi-cancer early detection testing, given its significance for population screening, is also briefly discussed. Liquid biopsy techniques offer promising avenues for improving PDAC diagnosis, prognostication, and management. In particular, proteomics shows considerable potential, yet further research is needed to validate existing findings and comprehensively explore the proteome using an unbiased approach.

## 1. Introduction

Pancreatic cancer is a devastating malignancy of concern due to its late diagnosis and high mortality [1]. Pancreatic ductal adenocarcinoma (PDAC) accounts for over 85% of pancreatic malignancies [2] and has a terrible prognosis due to its aggressiveness and treatment-refractory nature [3]. This is reflected by its dismal overall 5-year survival rate of 12.5% in Australia [4], and a median survival rate of approximately 7 months after diagnosis [5]. Unlike most other malignancies, both the incidence and mortality rate of PDAC continue to rise, and it is expected to become the second leading cause of cancer-related death worldwide by 2030 [6]. Thus, significant improvements in patient diagnosis and management are urgently needed.

Diagnosis of PDAC typically occurs late in the disease course, with 80% of patients reporting unresectable, locally advanced, or metastatic disease at diagnosis [7,8]. Surgical excision remains the single most effective, and only potentially curative treatment option for PDAC. Patient prognosis is inversely correlated with tumour stage, with a 5-year survival rate as high as 80.4% for stage I disease [8]. However, most patients present with advanced disease and hence suffer a worse prognosis [9]. This highlights the need to improve early-stage PDAC diagnostics, ideally detecting disease through asymptomatic screening. However, due to the overall low incidence of PDAC, general population-based screening is currently not recommended [10]. Yet, there is a window of opportunity for earlier diagnosis in asymptomatic patients, particularly in the higher-risk groups such as those with hereditary pancreatitis, chronic pancreatitis, a strong family history of PDAC, and late new-onset diabetes [11]. Despite concerted efforts, there remains no validated diagnostic or screening test that is ready for large-scale early detection of PDAC.

In the current treatment paradigm for PDAC, chemotherapy regimen selection typically follows institutional evidence-based guidelines without incorporating the use of predictive or prognostic biomarkers to guide clinical decision-making [12]. Carbohydrate antigen 19-9 (CA19-9) is presently the only biomarker utilised for PDAC prognostication, but has notable limitations, failing to diagnose 35% of patients with resectable PDAC [13]. Its implementation as a tool for screening and diagnosis is impeded by low sensitivity of approximately 80%, with poorer performance for earlier stage disease [14], and limited specificity of approximately 82% due to elevation in inflammatory conditions, biliary pathologies, and non-pancreatic malignancies [15]. With regards to PDAC prognostication, several surgical classification guidelines have recommended incorporating CA19-9 into the selection paradigm for borderline resectable pancreatic cancers (BRPC). Such guidelines utilise CA19-9 cut-offs ranging from 150 to 500 U/mL to select candidates with ‘high-risk’ characteristics of metastatic disease for pre-operative neoadjuvant chemotherapy approach prior to resection [16,17,18]. However, CA19-9 findings are generally only interpreted in combination with other investigations, such as imaging, and hence there is ongoing need for more reliable biomarkers for PDAC screening, diagnosis, and prognostication [17,19,20].

There is now growing interest in liquid biopsies as a means to survey tumour-derived biomarkers in peripheral blood, urine, and other biological fluids, such as peritoneal washings, in order to remotely assess disease burden. These methods boast the benefits of being non-invasive, relatively simple, and can safely be repeated multiple times to obtain longitudinal data [21]. This approach addresses the limitations of detecting early-stage PDAC, including diagnosing small tumours that are undetectable by radiological imaging and that are too small to biopsy by fine-needle aspiration (FNA). Liquid biopsies also overcome limitations of restricted tumour tissue availability and hurdles in obtaining specimens suitable for molecular analysis. Due to the hypocellular and stromal-rich nature of PDAC tumours [22], technical issues of insufficient tumour sampling or limited tumour cellularity are frequently encountered when attempting to perform molecular profiling from endoscopically obtained FNA cell block specimens [23].

Identification of novel biomarkers for PDAC holds immense potential to not only improve early detection and diagnosis, but also to support prognostication and guide patient management [24]. The applications of such techniques have been demonstrated for other cancer types, such as lung and breast cancer [25,26], but clinical applications in PDAC have only recently emerged. Data now demonstrate the feasibility of liquid biopsy techniques to guide adjuvant chemotherapy selection for early-stage PDAC, [27] as well as serial biopsy monitoring for predicting treatment response and prognosis [28].

**Figure 1:**
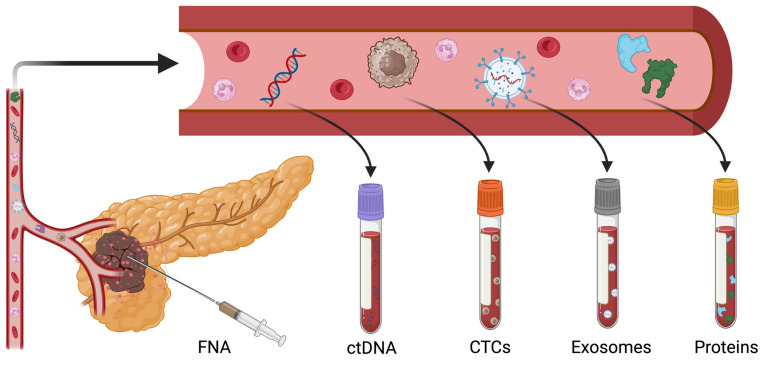
PDAC sampling by traditional tissue biopsy and liquid biopsy techniques. Current diagnostic workup for PDAC generally involves initial imaging such as computed tomography or magnetic resonance abdominal imaging, followed by confirmatory histopathological analysis of endoscopic ultrasound-guided fine-needle aspirates. Liquid biopsies present an alternative means to survey tumour-derived biomarkers accessible in peripheral blood, including circulating tumour DNA, circulating tumour cells, circulating tumour exosomes, and circulating free tumour-derived proteins (proteomics). Abbreviations: FNA, fine-needle aspirate; ctDNA, circulating tumour DNA; CTCs, circulating tumour cells. Schematic not to scale. Created with BioRender.

Biomarker technologies investigated for the purpose of liquid biopsy of PDAC include circulating tumour DNA (ctDNA), circulating tumour cells (CTCs), and circulating tumour exosomes [29]. More recently, proteomics has emerged as a promising alternative liquid biopsy option, but remains poorly characterised in pancreatic cancer. This review will examine each of these four technologies in turn, as shown in Figure 1, scrutinising recent developments and comparing their clinical utility in PDAC.

## 2. Methods

A systematic search of online database Ovid MEDLINE was performed in February 2024. Search keywords and Medical Subject Headings were related to pancreatic cancer and liquid biopsy techniques of interest to this study. The Boolean ‘OR’ operator was used to group related terms, and these groups were then combined using the Boolean ‘AND’ operator. This search returned 3147 articles.

**Figure 2:**
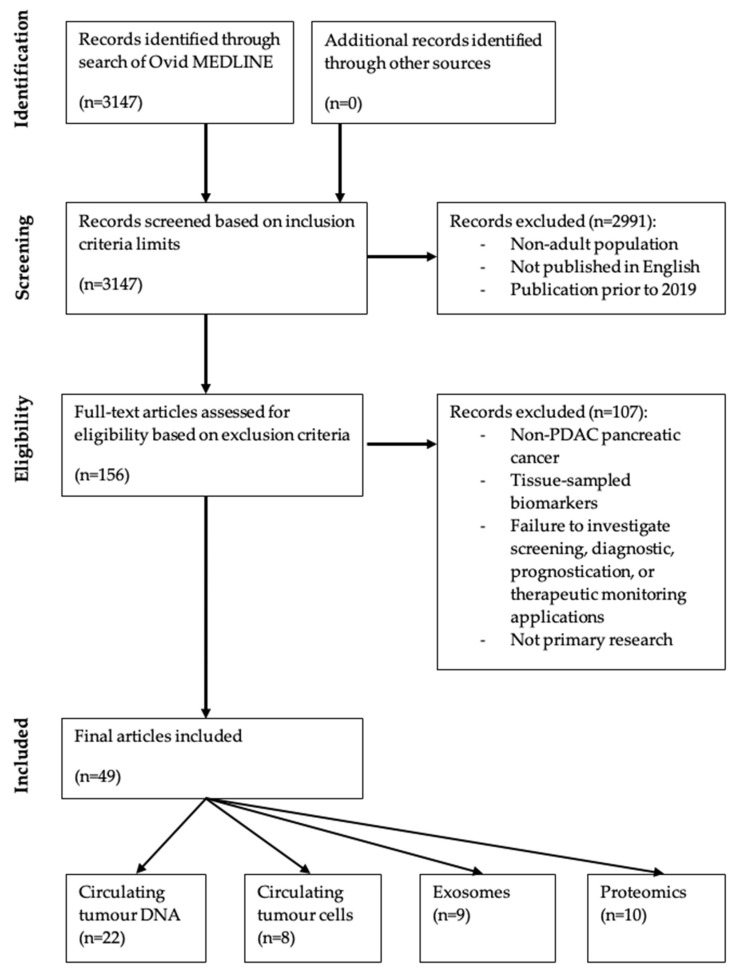
PRISMA diagram depicting literature search strategy. The Identification stage initially returned 3147 papers. The Screening and Eligibility stages excluded 2991 papers and 107 papers, respectively. Of the included forty-nine papers, twenty-two pertained to circulating tumour DNA, eight to circulating tumour cells, nine to exosomes, and ten to proteomics.

**Figure A1:**
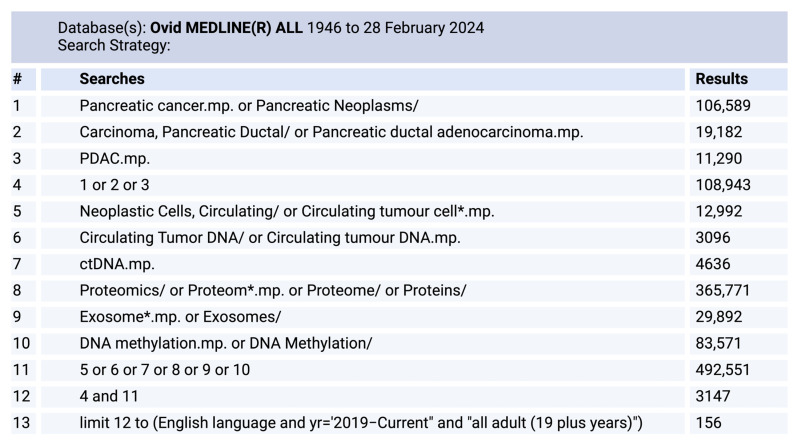
Ovid MEDLINE Search Strategy. Formatting consistent with Ovid syntax. Created with BioRender.

Limits were applied to the initial search strategy to meet the following inclusion criteria: (1) investigated adults only (defined as age ≥ 19 years on Ovid); (2) article available in English; and (3) article published 2019–present, given a major literature review of the topic was published in 2019 [29]. This limited the search results to 156 papers, which were then individually appraised for relevance. Papers were excluded based on the following exclusion criteria: (1) examination of non-PDAC pancreatic cancer; (2) examination of tissue-sampled biomarkers instead of peripheral fluids; (3) failure to investigate screening, diagnostic, prognostication, or therapeutic monitoring applications of the biomarker; and (4) case reports, letters, editorials, or reviews. The final number of accepted articles was 49. The PRISMA flow diagram outlining the literature search is depicted in Figure 2. The literature search strategy (prior to manual application of exclusion criteria) is described in Figure A1.

## 3. Discussion

### 3.1. Circulating Tumour DNA

Circulating tumour DNA (ctDNA) is a fraction of cell-free DNA (cfDNA) consisting of fragmented tumour-derived DNA shed from primary tumours or metastases and found in plasma and urine [30]. Methods of ctDNA analysis generally involve either sequencing of tumour-specific mutations or detecting aberrant DNA methylation patterns [31]. Additionally, there are two broad approaches for investigating ctDNA depending on whether the genetic aberrations present in the primary tumour are known or not, termed tumour-informed and tumour-agnostic techniques, respectively. In tumour-agnostic studies, ctDNA samples are analysed without prior examination of tumour tissue, and therefore target frequently mutated genes such as *KRAS*, or epigenetic changes such as hypermethylation in the promotor regions of tumour suppressor genes [32]. This approach is generally used in the context of cancer screening, and molecular diagnostic testing of patients with unresectable solid tumours, with next generation sequencing (NGS)-based assays commonly employed in such studies [33]. Conversely, tumour-informed approaches utilise sequencing data obtained from patient tumour tissue to create personalised gene panels that can be applied to ctDNA analysis [34]. Applications include detection of minimal residual disease or recurrence following curative-intent surgery, and monitoring of patient-specific therapeutic responses. Methods that adopt this tumour-informed approach include targeted sequencing platforms modified with molecular barcoding methods and error-suppression algorithms, such as Safe-SeqS, as well as polymerase chain reaction (PCR)-based platforms, such as droplet digital PCR (ddPCR) [35]. Tumour-informed studies have intrinsically higher sensitivity in detecting disease due to their application in only patients with previously known tumours [34]. Overall, there is much ongoing interest exploring the value of ctDNA analysis in guiding PDAC diagnosis, prognostication, and management. Details of the 22 identified studies pertaining to ctDNA analysis are outlined in Table 1.

The diagnostic utility of *KRAS*-mutated ctDNA in PDAC patient plasma has recently been explored by several groups, reporting varying sensitivities based on different assays. Among newly diagnosed patients, published sensitivities include 37% [36], 48% [37,38], 49% [39], and 62% [40]. Others have trialled panels of cancer-related genes and have described sensitivities of 38% [41], 44% [42], 45% [43], 50% [44], 66% [45], 68% [46], and 75% [47]. Additionally, Terasawa et al. examined ctDNA in PDAC patient urine and reported a sensitivity of 48% whilst also identifying that renal function may act as a potential confounder [38]. These studies frequently identified mutated *KRAS* and *TP53* as the most common ctDNA fragments in PDAC patient plasma, consistent with their role in PDAC oncogenesis [48], yet these low reported sensitivities somewhat undermine the feasibility of ctDNA-based diagnostic testing. Considering MacGregor-Das et al. identified *KRAS* ctDNA-positivity in just 31% of patients with stage I/II disease [36], whether ctDNA alone will contribute a major role to asymptomatic PDAC screening in the future is also yet to be determined. Furthermore, the positive predictive value of mutational ctDNA analysis is hampered by false positive results arising from multiple sources. *KRAS*-mutated ctDNA is carried by some healthy individuals, as noted in the preceding study [36], which is consistent with prior understanding that non-cancerous circulating lymphoid and myeloid cells can accumulate confounding somatic mutations [49]. False positive results may also arise ex vivo due to sequencing read errors [50] and inadvertent chemical modification of ctDNA, such as by cytosine deamination [36].

As an alternative means for diagnostic testing, ctDNA hypermethylation patterns have been described for several genes. Pietrasz et al. identified *HOXD8* and *POU4F1* hypermethylation had a sensitivity of 56.8% in a large cohort of 372 patients with metastatic PDAC [51]. Another group found *SPARC* and *NPTX2* hypermethylation can distinguish PDAC from healthy controls and patients with chronic pancreatitis [52]. Alternatively, *ZNF154* has been reported to discern cancer patients, including those with pancreatic, colorectal, and liver malignancies, from healthy controls [53]; however, such testing inherits intrinsic limitations to specificity. Other studies have combined methylation analysis with other metrics to improve test performance. For example, Shinjo et al. found the sensitivity of a five gene panel containing *ADAMTS2*, *HOXA1*, *PCDH10*, *SEMA5A*, and *SPSB4* improved from 49% to 68% when combined with testing for *KRAS*-mutated ctDNA [54]. Similarly, Fujimoto et al. reported an increase in sensitivity from 50.9% to 85.5% for a *RUNX3*-based assay, once combined with CA19-9 measurement [55]. However, despite these increases, the sensitivities of such tests remain generally low and thus have limited clinical utility.

Perhaps more promising is the prognostic potential of ctDNA detection. Several groups found significant associations between mutant *KRAS*–ctDNA positivity and inferior prognosis across the spectrum of disease, including pre-operative patients, those with residual disease post-resection, and those with metastatic disease. Specifically, positive mutant *KRAS*–ctDNA status has been associated with earlier tumour recurrence, greater metastatic burden, and shorter median progression-free survival (mPFS) and median overall survival (mOS) [37,39,40,41,56,57,58]. These findings were also mirrored in gene panel studies [43,44,45,46,47]. Only one group reported no association between pre-operative *KRAS*–ctDNA status and overall prognosis [59]. There are considerably fewer publications exploring methylation-based prognostication, yet they largely support these mutational studies. Singh et al. found *SPARC* and *NPTX2* hypermethylation correlate with disease stage and poorer survival [52], whilst Pietrasz et al. identified *HOXD8* and *POU4F1* as independent prognostic markers for mPFS and mOS [51], and Wang et al. found that total unfractionated cfDNA also correlates with poorer patient prognosis [60]. Overall, these studies support a prognostic role for ctDNA analysis in PDAC patients.

Furthermore, some research now links ctDNA testing to predictive therapeutic utility. Decreases in mutant *KRAS*–ctDNA titres within six months of commencing first-line palliative chemotherapeutics has been correlated with better treatment response [59]. Similarly, Bachet et al. report decreases in ctDNA among patients receiving eryaspase therapy correlates with better drug efficacy [46]. Wei et al. also report that increases and decreases in ctDNA methylation for a variety of genes correlate with chemotherapy resistance and responsiveness, respectively [45]. These early studies show the potential for ctDNA analysis to guide patient management via selection of effective therapeutics; however, further studies in larger cohorts will be required for clinical validation. Furthermore, relatively few studies utilising tumour-informed ctDNA analysis have been published, and such studies would further support these findings.

**Table 1:** Characteristics of ctDNA studies identified for analysis.

Citation	Patients		Applications Explored				Methodology			
	N	Disease Descriptor	Study Aim	Diagnosis (Sensitivity, Specificity)	Prognostic	Predictive	Agnostic/ Informed	Broad Method	Detection Technology	Gene(s) Examined
Bachet et al., 2020 [46]	113	Stage IV	To evaluate the prognostic and predictive value of ctDNA from plasma samples during a randomised phase II trial which has assessed eryaspase efficacy in patients with advanced PDAC.	Yes (68.1, NR)	Yes	Yes	Agnostic	Mutation	*AmpliSeq* colon and lung cancer panel V2 (NGS)	22
Cheng et al., 2020 [58]	210	Stage III–IV	To evaluate the *KRAS* mutation status in ctDNA and circulating T cell subsets in a cohort of advanced pancreatic cancer patients.	No	Yes	No	Agnostic	Mutation	ddPCR	*KRAS*
Chung et al., 2021 [42]	77	Stage IV	To characterise the mutational landscape of patients with metastatic PDAC who received blood-based molecular profiling.	Yes (44.1, NR)	No	No	Agnostic	Mutation	*Guardant-360* (NGS)	83
Fujimoto et al., 2021 [55]	55	Stage I–IV	To evaluate the sensitivity and specificity of serum DNA testing of methylated *RUNX3* by the CORD assay for the detection of PDAC.	Yes (50.9, 93.5)	No	No	Agnostic	HM	CORD assay	*RUNX3*
Groot et al., 2019 [39]	59	Pre- and post-operative	To perform analytical and clinical validation of a *KRAS* ctDNA assay in a certified clinical laboratory.	Yes (49.2, NR)	Yes	No	Agnostic	Mutation	ddPCR	*KRAS*
Guo et al., 2020 [41]	113	Pre-operative	To explore the clinical value of hotspot mutations in resectable PDAC patients.	Yes (38.1, NR)	Yes	No	Agnostic	Mutation	NGS	50
Hussung et al., 2021 [37]	25	Post-operative	To identify associations between mutant cf*KRAS* and CA19-9 dynamics and clinical outcome post resection.	Yes (48.0, NR)	Yes	No	Agnostic	Mutation	ddPCR	*KRAS*
Lee et al., 2019 [40]	42	Pre-operative	To evaluate the feasibility and clinical utility of ctDNA analysis to inform adjuvant therapy decision making.	Yes (62.2, NR)	Yes	No	Informed	Mutation	Safe-SeqS assay (PCR)	*KRAS*
Li et al., 2021 [56]	105	Pre-operative	To establish a scoring system for preoperative screening of resectable PDAC patients.	No	Yes	No	Agnostic	Mutation	qPCR	*KRAS*
Macgregor-Das et al., 2020 [36]	67	Screening and pre-operative	To utilise enzymatic pretreatment of plasma DNA followed by digital NGS to detect hotspot mutations in *KRAS* and *GNAS* in patients with pancreatic cancer.	Yes (36.5, 92.0)	No	No	Agnostic	Mutation	Digital NGS	*KRAS* *GNAS*
Miller et al., 2021 [53]	17	Stage I–IV	To assess the effectiveness of hypermethylation at the CpG island of *ZNF154* for use in a blood-based cancer detection assay.	Yes (NR, NR)	No	No	Agnostic	HM	ddPCR	*ZNF154*
Patel et al., 2019 [47]	112	Stage I–IV	To assess the genomic landscape of ctDNA in patients with PDAC, using clinical-grade NGS to investigate the clinical implications.	Yes (69.6, NR)	Yes	No	Agnostic	Mutation	NGS	54–73
Pietrasz et al., 2022 [51]	372	Stage IV; CTx-naïve	To determine whether ctDNA is an independent factor for the prognostication of metastatic PDAC.	Yes (56.8, NR)	Yes	No	Agnostic	HM	ddPCR	*HOXD8* *POU4F1*
Shinjo et al., 2020 [54]	47	Stage I–IV	To identify effective DNA methylation markers for the diagnosis of PDAC.	Yes (49.0, 86.0)	No	No	Informed	HM	MBD-ddPCR	*HOXA1* *PCDH10* *ADAMTS2* *SEMA5A* *SPSB4*
Singh et al., 2020 [52]	65	Stage I–IV	To conduct absolute quantification of methylation in *SPARC*, *UCHL1*, *PENK*, and *NPTX2* genes and assess the respective methylation load for their ability to perform as non-invasive differentiating and prognostic marker(s) for PDAC.	Yes (NR, NR)	Yes	No	Agnostic	HM	qPCR	*SPARC* *UCHL1* *PENK* *NPTX2*
Strijker et al., 2020 [43]	58	Stage IV; CTx-naïve	To evaluate the capability of targeted sequencing using a custom pancreatobiliary NGS panel and of ddPCR to detect ctDNA in metastatic PDAC.	Yes (44.8, NR)	Yes	No	Agnostic	Mutation	NGS	8
Sugimori et al., 2020 [57]	21	Stage IV	To assess the dynamics of ctDNA in patients with advanced PDAC undergoing chemotherapy using dPCR.	No	Yes	No	Agnostic	Mutation	dPCR	*KRAS*
Terasawa et al., 2019 [38]	56	Stage II–IV	To analyse *KRAS* mutations in urine to investigate the potential of urine liquid biopsy in PDAC.	Yes (48.2, NR)	No	No	Agnostic	Mutation	ddPCR	*KRAS*
Uesato et al., 2020 [44]	104	Stage IV	To evaluate circulating tumor DNA as a tumor biomarker to prognosticate pancreatic cancer.	Yes (50.0, NR)	Yes	No	Agnostic	Mutation	*Oncomine* colon cfDNA assay (NGS)	14
Wang et al., 2021 [60]	97	Pre-operative	To evaluate the role of cfDNA in PDAC of the pancreatic head.	No	Yes	No	-	Total cfDNA	qPCR	-
Watanabe et al., 2019 [59]	78	Pre- and post- CTx and/or surgery	To evaluate the significance of sequentially assessing *KRAS* ctDNA levels through longitudinal monitoring.	No	Yes	Yes	Informed	Mutation	ddPCR	*KRAS*
Wei et al., 2019 [45]	38	Receiving palliative CTx	To explore the application of cfDNA profiling in monitoring tumor burden in patients with PDAC.	Yes (65.8, 100)	Yes	Yes	Agnostic	Mutation	NGS	560

Notes: N denotes number of PDAC patients included in study only. Most appropriate descriptor of disease has been applied, including AJCC (8th) disease stage or resection state. Diagnostic sensitivity is reported relative to healthy controls, without stratification by disease stage, and without combination with other metrics (e.g., CA19-9). Genes examined presented as names if ≤5 reported, or number of genes in panel if >5 reported. All numbers reported to three significant figures, wherever possible. Abbreviations: ctDNA, circulating tumour DNA; cfDNA, cell-free DNA; NR, not reported (or unable to be deconvoluted from reported data); HM, hyper-methylation; NGS, next-generation sequencing; PCR, polymerase chain reaction; dPCR, digital PCR; ddPCR, droplet digital PCR; CORD, combined restriction digital PCR; qPCR, quantitative PCR; MBD-ddPCR, methyl-CpG binding ddPCR; CTx, chemotherapy.

### 3.2. Circulating Tumour Cells

Circulating tumour cells (CTCs) are malignant cells that can be isolated from the peripheral blood of cancer patients, providing some measure of tumour burden [61]. Several recent studies have correlated the presence and/or quantity of CTCs with clinical data in PDAC. These studies have historically analysed the total population of a patient’s CTCs, but, more recently, research is emerging exploring CTC subpopulations. Details of the eight identified studies pertaining to CTC analysis are delineated in Table 2.

Studies investigating the diagnostic utility of assessing total CTCs have been somewhat discouraging. A large study of 106 PDAC patients reported a trend of increasing sensitivity with disease stage, with CTC-positivity rates ranging from just 37.5% for stage II to 100% for stage IV patients [62]. Discounting disease stage, several groups have published findings of overall CTC positivity in 75% [63], 76% [64], 77% [65], and 79% [66] of PDAC patients, whilst Park et al. have reported 33% positivity among patients post-resection [67]. Each of these studies also explored the prognostication capacity of total CTC analysis, with variable findings. Many groups correlated CTC-positivity with poorer prognosis, including increased recurrence risk and shorter mPFS [62,63,64,66,67], whilst Semaan et al. found no correlation between total CTC count and any clinicopathological variables in their study of 74 PDAC patients [65].

More recently, these groups and others have identified subpopulations of CTCs related to epithelial–mesenchymal transition (EMT) phenotypes. Three subpopulations are commonly identified based on expression of epithelial markers (generally cytokeratin and EpCAM), mesenchymal markers (generally vimentin and Twist), or both (termed ‘mixed’ CTCs). With respect to diagnostic testing, detection of mesenchymal CTCs in conjunction with CA19-9 testing was found to hold some diagnostic potential, with an area under the receiver operator curve (AUROC) of 0.968 [64]. Similarly, isolation of an unrelated CTC subpopulation based on folate receptor expression among patients with pancreatic malignancy achieved an AUROC of 0.944 when combined with CA19-9 [68]. Despite these improvements in diagnostic potential, explorations of subpopulation prognostic capacity have yielded variable results. Some groups have found that a higher CTC proportion exhibiting a mixed phenotype is correlated with advanced disease stage [66], earlier recurrence, and worse mPFS and mOS [65]. Alternatively, Xing et al. reported that both mixed and mesenchymal CTC-positivity were associated with shorter mPFS [62], whilst Zhu et al. identified that only mesenchymal CTCs were linked to a worse prognosis [63]. These data broadly align with prior research that has established CTCs with a mesenchymal or mixed phenotype show greater invasiveness [69], and thus may be linked to a poorer prognosis. However, a small study of 21 patients by Gasparini et al. reported no significant relationship between mesenchymal CTCs and mPFS or mOS [70].

Considered together, these data reveal persistent limitations in the utility of CTC analysis for PDAC diagnosis and prognostication. In particular, low sensitivity in detecting earlier stage disease [62] renders prospects of developing CTC-based screening impractical at this time. Moreover, conclusions reached by these studies in identifying clinicopathological correlates for prognostication exhibit stark inconsistencies. This lack of consensus hinders the development of reliable CTC-based testing due to lack of established thresholds or endpoints.

Variability in reported findings may be, in part, attributable to differences in study methodology. Briefly, there are two fundamental techniques for CTC isolation based on either biomolecule expression or physical properties, which are known to somewhat underestimate and overestimate results, respectively [29]. As such, some variability in conclusions drawn by the reviewed articles may be explained by differences in CTC detection techniques, given some studies utilised expression-based assays (such as immunomagnetic separation), whilst others used morphology-based assays (such as dielectrophoretic-field flow fractionation). By extension, the intrinsic inaccuracies of the testing modalities, coupled with the rarity and low yield of isolating CTCs from patient blood [71], render CTC-based test development difficult.

**Table 2:** Characteristics of CTC studies identified for analysis.

Citation	Patients		Applications Explored			Methodology		
	N	Disease Descriptor	Study Aim	Diagnosis [AUROC] or (Sensitivity, Specificity)	Prognostic	Total/ Subpops	Biomolecule/Physical	Isolation Technology
Cheng et al., 2020 [68]	45 ^a^	Stage I–IV	To investigate the diagnostic value of folate receptor-positive CTCs in distinguishing pancreatic cancer from benign pancreatic disease.	Yes [0.837]	No	Folate-receptor positive	Biomolecule	LT-PCR
Gasparini-Junior et al., 2019 [70]	21	Stage II–IV	To correlate the number of CTCs in the peripheral blood of patients with locally advanced or metastatic pancreatic tumors, and the protein expression involved in EMT in CTCs with clinical characteristics, mPFS, and mOS.	No	Yes	Total and EMT subpops	Physical	ISET
Park et al., 2021 [67]	36	Post-operative	To determine whether the preoperative presence of CTCs is associated with the overall survival and recurrence-free survival in patients with PDAC.	Yes (33.3, NR)	Yes	Total only	Physical	*CD-PRIME*
Semaan et al., 2021 [65]	74	Stage I–IV	To perform comprehensive phenotypic characterisation of CTCs and their clinical significance in a longitudinal cohort of PDAC patients.	Yes (76.8, NR)	Yes	Total and EMT subpops	Physical	DEP-FFF
Wei at al., 2019 [64]	100	Stage I–IV	To determine whether cell-surface vimentin could be a biomarker to isolate CTCs in PDAC.	Yes (76.0, 97.4)	Yes	Total and EMT subpops	Biomolecule	*CytoQuest CR*
Xing et al., 2022 [62]	106	Stage II–IV	To explore the relationship between CTCs or T lymphocyte subsets and prognosis in patients with pancreatic cancer.	Yes (75.5, NR)	Yes	Total and EMT subpops	Physical	*CanPatrol CTC*
Zhao et al., 2019 [66]	107	Stage I–IV	To evaluate the clinical properties of three CTC subpopulations undergoing EMT in PDAC patients.	Yes (78.5, NR)	Yes	Total and EMT subpops	Physical	*CanPatrol CTC*
Zhu et al., 2021 [63]	40	Pre-operative	To determine the prognostic significance of CTCs expressing Krüppel-like factor 8 and vimentin in pancreatic cancer.	Yes (75.0, NR)	Yes	Total and EMT subpops	Biomolecule	Immuno-magnetic separation

Notes: N denotes number of PDAC patients included in study only. Most appropriate descriptor of disease has been applied, including AJCC (8th) disease stage or resection state. Diagnostic performance is reported as AUROC where possible, or sensitivity and specificity; reported relative to healthy controls, without stratification by disease stage, and without combination with other metrics (e.g., CA19-9). All numbers reported to three significant figures, wherever possible. Abbreviations: CTC, circulating tumour cell; AUROC, area under receiver operator curve; subpops, subpopulations; EMT, epithelial–mesenchymal transition; NR, not reported (or unable to be deconvoluted from reported data); PCR, polymerase chain reaction; LT-PCR, ligand-targeted PCR; ISET, isolation by size of tumour cells; DEP-FFF, dielectrophoretic field-flow fractionation. ^a^ Study population included 4% patients with intraductal papillary mucinous neoplasms, and data could not be deconvoluted to examine PDAC only.

### 3.3. Circulating Tumour Exosomes

Circulating tumour exosomes are extracellular vesicles released by cells, including cancer cells, into peripheral blood [72]. The content of an exosome reflects that of its parent cell, and typically includes many bioactive molecules such as nucleic acids, proteins, and lipids [29]. Consequently, recent studies have sought to explore the diagnostic and prognostic potential of exosome analysis in PDAC. Details of the nine identified studies pertaining to exosome analysis are outlined in Table 3.

Recent developments have examined several types of nucleic acids in circulating tumour exosomes. With respect to PDAC diagnostics, expression of long non-coding RNA HULC has been identified as being significantly elevated in patient plasma compared to healthy controls and intraductal papillary mucinous neoplasm (IPMN) patients [73]. Similarly, Kitagawa et al. reported messenger RNAs WASF2 and ARF6, and small nucleolar RNAs SNORA74A and SNORA25, can each distinguish pancreatic cancer patients from healthy controls with an AUROC of >0.9, including for patients with stage I/II disease [74]. Furthermore, a suite of microRNAs capable of distinguishing PDAC from related pathologies has been published by Vicentini et al., with nine, twenty, and twenty-two miRNAs found to differ significantly in PDAC, compared to chronic pancreatitis, IPMN, and ampulla of Vater carcinoma, respectively. Moreover, regarding prognostication, this group also identified a signature of 11 miRNAs able to differentiate localised PDAC from metastatic disease [75]. Thus, nucleic acid analysis holds potential for PDAC diagnosis and prognostics; however, these studies cumulatively examined just 102 patients, and thus validation in larger cohorts is warranted. In like manner, one study investigating exosomal lipids has proved similar merit, identifying approximately 270 lipids that are significantly dysregulated in pancreatic cancer patients, and that specifically lysophosphatidylcholine (22:0), phosphatidylcholine (P-14:0/22:2), and phosphatidylethanolamine (16:0/18:1) are significantly correlated with tumour stage and mOS [76]. However, this study suffers the similar limitation of examining just 22 patients.

Explorations of exosomal protein expression have yielded some promising results, and in larger cohorts. Diagnostic studies by Wei et al. identified exosomal Eph receptor A2 (EphA2) levels were significantly elevated among 244 pancreatic cancer patients. Indeed, AUROC values for serum exo-EphA2 diagnostic testing were 0.94 and 0.92 against healthy controls and benign pancreatic disease, respectively. These values decreased slightly when examining early PDAC (stages I/II) to 0.92 and 0.90, respectively, yet combining exo-EphA2 with CA19-9 measurement then raised AUROC to 0.96 against healthy controls [77,78]. These authors also found serum exo-EphA2 levels were significantly higher among patients with advanced disease (stages III/IV) compared to those with earlier stage disease, providing some indication of prognostic value. Whilst encouraging, these data would benefit from replication by an independent research group. Xiao et al. achieved similar diagnostic efficacy using a panel of serum exosomal glypican-1, exosomal CD82, and CA19-9, publishing an AUROC of 0.942 [79]. Conversely, another group examining exosomal c-Met expression reported significantly higher expression levels among PDAC patients compared to benign disease, but poor diagnostic capacity with a sensitivity of 70% and specificity of 85% [80]. Regarding prognostication, these authors did, however, note exosomal c-Met-positivity and PD-L1-positivity correlated with shorter mOS after surgery. Interestingly, Giampieri et al. identified that a higher quantity of exosomal EpCAM was associated with poorer response to palliative chemotherapy and shorter mOS [81]. This is seemingly at odds with recent research into CTC-based PDAC prognostics, as discussed previously, whereby several groups identified CTCs adopting a mesenchymal EpCAM-negative phenotype were associated with poorer prognosis, consistent with the invasive characteristics associated with mesenchymal tumour cells [69]. However, this group also identified increases in EpCAM expression during chemotherapy were instead associated with longer mPFS [81]. Such inconsistencies may be, in part, explained by the small sample size of 19 for this study. Overall, much like nucleic acid- and lipid-based research, exosomal proteins analysis offers generally good test characteristics for the diagnosis and prognostication of PDAC. However, validation of the discussed findings must be conducted in larger cohorts, with a goal of corroboration between multiple research groups for the studied disease markers.

**Table 3:** Characteristics of circulating tumour exosome studies identified for analysis.

Citation	Patients		Applications Explored			Methodology		
	N	Disease Descriptor	Study Aim	Diagnosis [AUROC] or (Sensitivity, Specificity)	Prognostic	Biomolecule Class	Analysis Technique	Molecule(s) Examined
Giampieri et al., 2019 [81]	19	Receiving palliative CTx	To analyse the contents of circulating exosomes in patients with pancreatic cancer who received palliative CTx.	No	Yes	Protein	ELISA	10
Kitagawa et al., 2019 [74]	27	Stage I–III	To assess the utility of several serum mRNAs and snoRNAs as diagnostic markers for differentiating PDAC patients from control patients without pancreatic disease.	Yes [0.883] ^a^	Yes	mRNA snoRNA	RT-qPCR	4 (mRNA) 5 (snoRNA)
Lux et al., 2019 [80]	55	Stage I–IV	To assess whether pancreatic carcinomas release exosomes which express c-Met and PD-L1, and whether the detection of such expression in serum has diagnostic or prognostic meaning for the affected patients.	Yes (70, 85)	Yes	Protein	Flow cytometry	c-Met PD-L1
Takahashi et al., 2020 [73]	20	Stage II–IV	To identify lncRNAs involved in the EMT pathway and investigate their functional roles during PDAC cell invasion and migration.	Yes [0.92] ^b^	Yes	lncRNA	dPCR	HULC
Tao et al., 2019 [76]	22	Post-operative	To identify the possible prognostic or diagnostic metabolite biomarkers in the serum exosome of PDAC patients.	Yes (NR, NR)	Yes	Lipid	LC-DDA-MS	270
Vicentini et al., 2020 [75]	55	Stage I–IV	To identify circulating miRNAs able to discriminate different histotypes of pancreatobiliary neoplasms.	Yes (NR, NR)	Yes	miRNA	*NanoString*	22
Wei et al., 2020 [77]	40	Stage I–IV	To understand the mechanism underlying pancreatic cancer metastasis to identify novel biomarkers.	Yes (NR, NR)	Yes	Protein	ELISA	EphA2
Wei et al., 2020 [78]	204	Stage I–IV	To evaluate serum Exo-EphA2 as a potential diagnostic biomarker in pancreatic cancer.	Yes [0.94]	Yes	Protein	ELISA	EphA2
Xiao et al., 2020 [79]	27	NR	To establish a simple and efficient standard method for the detection and analysis of exosomal GPC1 protein to explore screening value in Chinese patients with pancreatic cancer.	Yes [0.869] ^c^	No	Protein	Flow cytometry	GPC1 CD82

Notes: N denotes number of PDAC patients included in study only. Most appropriate descriptor of disease has been applied, including AJCC (8th) disease stage or resection state. Diagnostic performance is reported as AUROC where possible, or sensitivity and specificity; reported relative to healthy controls, without stratification by disease stage, and without combination with other metrics (e.g., CA19-9). Molecule(s) examined presented as names if ≤5 reported, or number of molecules if >5 reported. All numbers reported to three significant figures, wherever possible. Abbreviations: AUROC, area under receiver operator curve; NR, not reported (or unable to be deconvoluted from reported data); RNA, ribonucleic acid; mRNA, messenger RNA; snoRNA, small nucleolar RNA; lncRNA, long non-coding RNA; miRNA, micro-RNA; ELISA, enzyme-linked immunosorbent assay; PCR, polymerase chain reaction; RT-qPCR, reverse transcription quantitative PCR, dPCR, digital PCR; LC-DDA-MS, liquid chromatography data-dependent acquisition mass spectrometry; CTx, chemotherapy. ^a^ AUROC reported as median of published values for each of nine independent markers. ^b^ AUROC reported as relative to both healthy controls and IPMN patients combined. ^c^ AUROC reported as mean of published values for two independent markers.

### 3.4. Proteomics

The application of free protein-based testing for PDAC is now an emerging field for liquid biopsies that boasts promising early results. Proteomic studies to date have mostly analysed the proteome of patient plasma and sera, enabling an in-depth dynamic assessment of how genes are expressed, and protein–protein interactions. Compared to targeted genomic analysis, which is a static assessment of mutational tumour DNA, the systemic large-scale analysis of proteins offers increased technical capability to detect rarer alterations, functional changes, as well as a better understanding of the nature of protein-drug interactions. Details of the 10 identified studies pertaining to analysis of circulating proteins are described in Table 4.

One large study of 401 PDAC patients by Kim et al. investigating the diagnostic capabilities of a 14-marker proteomics panel achieved AUROC values of 0.977, 0.953, and 0.928, for one exploratory and two independent validation cohorts, respectively [82]. This study involved many control populations, including healthy volunteers, patients with benign pancreatic lesions, and patients with other types of cancer. The diagnostic performance of this panel significantly outperformed CA19-9 alone, yet together these metrics were able to reach an impressive AUROC of 0.989. Similarly, Wu et al. identified PROZ and TNFRSF6B serum levels could significantly distinguish PDAC patients from healthy controls and benign pancreatic pathologies, achieving an AUROC of 0.971 overall and 0.913 for stage I disease, when combined with CA19-9 [83]. A PDAC risk prediction model, developed by Kartsonaki et al., based on known risk factors such as age, smoking, and alcohol consumption, significantly improved after factoring in serum concentrations of several proteins, including angiopoietin-2, monocyte chemotactic protein-3, interleukin-6, and interleukin-18, with a significant increase in weighted C-statistic from 0.85 to 0.99 [84]. Smaller studies have achieved varying diagnostic success. For example, one study of twenty-two patients identified a panel of seven peptides able to distinguish PDAC from healthy controls with 93% sensitivity [85], whilst another group identified a panel of eight proteins able to discriminate early PDAC from healthy individuals with an AUROC of 0.81–0.85 [86]. Comparably, Deutsch et al. examined the salivary proteome of fifteen male PDAC patients and found a panel of five proteins (cytokeratin-14, cytokeratin-16, cytokeratin-17, lactoperoxidase, and peptidyl-prolyl cis–trans isomerase B) that could discern patients from healthy controls with an AUROC of 0.91 [87].

With respect to proteomics based PDAC prognostication, few studies have yet been published, and these generally examine relatively small cohorts. Sahni et al. identified lower plasma levels of protein tyrosine phosphatases PTPRB and PTPRM were associated with poorer prognosis in a study of 12 patients [88], whilst Holm et al. identified a panel of 31 proteins, including several enzymes and complement proteins, which significantly differed between 21 PDAC patients with long or short mOS [89]. Similarly, elevated concentration of plasma fucosylated SERPINA1 has been significantly correlated with disease stage and shorter mOS [90]. Furthermore, Rittman et al. reported that serum concentration of 23 proteins, including MAEA, could effectively distinguish PDAC patients with early versus late recurrence. This group further identified MAEA expression is significantly elevated by several PDAC organoid lines, and that organoid-derived MAEA levels could similarly discriminate early and late recurrence [91]. Therefore, whilst the development of proteomics-based PDAC testing is relatively novel, early studies have showed incredible potential for diagnostic utility, particularly in conjunction with CA19-9 testing, having reported the highest AUROC values of all related publications in the past five years. However, in a similar vein to circulating tumour exosomes, validation and corroboration of research findings between groups remains to be seen and will be required moving forwards. Furthermore, several of these studies analysed only pre-determined candidate proteins, and comprehensive explorations of the proteome among large and diverse PDAC cohorts remain limited. Regarding prognostication, relatively little evidence yet exists, and, similarly, larger exploratory and validation studies are needed. The relative limitations and strengths of the four PDAC biomarker modalities discussed herein are summarised in Table 5.

**Table 4:** Characteristics of proteomics studies identified for analysis.

Citation	Patients		Applications Explored			Methodology		
	N	Disease Descriptor	Study Aim	Diagnosis [AUROC] or (Sensitivity, Specificity)	Prognostic	Candidate Only	Analysis Technique	Protein(s) Examined
Deutsch et al., 2020 [87]	15	Stage III–IV	To identify and develop an early detection assay for pancreatic cancer based on candidate biomarkers in oral fluids.	Yes [0.91]	No	No—comp (oral fluids)	DM-LCT-MS	Cytokeratin-14 Cytokeratin-16 Cytokeratin-17 Lacto-peroxidase Peptidyl-prolyl cis–trans isomerase B
Duan et al., 2019 [85]	22	Stage I–III	To identify differentially expressed peptides involved in pancreatic cancer as potential biomarkers.	Yes (93.0, 94.6)	No	No—comp	LC-ESI-MS	7
Holm et al., 2020 [89]	21	Stage I, II, IV	To analyse the serum proteome of a small cohort of PDAC patients and compare the protein expression between patients with short- and long-term survival, in order to discover proteins that could be of value as new candidates for prognostic biomarkers.	No	Yes	No—comp	UPLC-UDMS	31
Kartsonaki et al., 2022 [84]	610	NR	To examine the prospective associations of >90 protein biomarkers with development of pancreatic cancer and to assess the extent to which they could help predict risk of a future diagnosis.	Yes (C-statistic) ^a^	No	Yes	*Olink*—immuno-oncology panel	92
Kim et al., 2021 [82]	401	Stage I–IV	To develop and validate a protein-based, multi-marker panel that provides superior PDAC detection abilities with sufficient diagnostic performance.	Yes [0.977]	No	Yes	MRM-MS	14
Rittmann et al., 2021 [91]	14	Pre-operative	To identify preoperative plasma protein biomarkers with the potential to predict early recurrence after resection of PDAC.	No	Yes	Yes	*Olink*—several panels	23
Sahni et al., 2020 [88]	12	Pre-operative	To identify potential prognostic biomarkers in plasma and isolated microparticles from PDAC patients.	No	Yes	No—comp	SWATH-MS	PTPRB PTPRM
Wu et al., 2021 [90]	71	Stage I–IV	To identify novel plasma glycobiomarkers of pancreatic cancer, with a view to analysing the glycoproteome of plasma samples from patients with non-metastatic and metastatic cancer and gallstones.	Yes (NR, NR)	Yes	No—comp (glycoproteome)	LC-MS	22
Wu et al., 2019 [83]	183	Stage I–IV	To identify proteomic changes in sera of pancreatic cancer patients, and subsequently evaluate the expression levels of these proteins to evaluate their potential as possible diagnostic markers.	Yes [0.966]	No	No—comp	LC-MS	PROZ TNFRSF6B
Yu et al., 2021 [86]	135	Stage I–IV	To identify plasma protein biomarkers for early detection of PDAC.	Yes [0.81–0.85] (2 cohorts) ^b^	No	Yes	*Olink*—oncology II panel	8

Notes: N denotes number of PDAC patients included in study only. Most appropriate descriptor of disease has been applied, including AJCC (8th) disease stage or resection state. Diagnostic performance is reported as AUROC where possible, or sensitivity and specificity; reported relative to healthy controls, without stratification by disease stage, and without combination with other metrics (e.g., CA19-9). Protein(s) examined refers to those explored in detail, and is presented as names if ≤5 reported, or number of proteins if >5 reported. All numbers reported to three significant figures, wherever possible. Abbreviations: AUROC, area under receiver operator curve; NR, not reported (or unable to be deconvoluted from reported data); comp, comprehensive; MS, mass spectrometry; DM-LCT-MS, dimethylation liquid chromatography tandem MS; LC-ESI-MS, liquid chromatography electrospray ionisation MS; UPLC-UDMS, ultra-performance liquid chromatography ultra-definition MS; MRM-MS, multiple reaction monitoring MS; SWATH-MS, sequential window acquisition of all theoretical mass spectra; LC-MS, liquid chromatography MS. ^a^ Diagnostic utility reported as significant improvement in C-statistic rather than AUROC or sensitivity/specificity. ^b^ AUROC reported as range of published values for two independent cohorts.

**Table 5:** Summary of relative limitations and strengths of biomarker modalities.

Modality	Limitations	Strengths
ctDNA	▪ Low diagnostic test sensitivity, especially in early disease; ▪ Limited studies exploring tumour-informed approaches.	▪ Good evidence for prognostication and predictive therapeutic response monitoring.
CTCs	▪ Low diagnostic test sensitivity, especially in early disease; ▪ Prognostic study discrepancies between published works; ▪ Analysis methods display intrinsic inaccuracies; ▪ Rarity and low CTC yield from patient blood samples.	▪ Highly specific (CTCs absent in healthy individuals); ▪ Evidence suggests sub-analysis of CTC subpopulations may improve test performance.
Exosomes	▪ Relative paucity of published papers to date; ▪ Low sample size for many recent explorations; ▪ Lack of validation and corroboration between authors.	▪ Good test performance for diagnosis and prognosis.
Proteomics	▪ Relative paucity of published papers to date; ▪ Prognostication not yet comprehensively explored; ▪ Lack of validation and corroboration between authors; ▪ Several studies to date explored only candidate proteins.	▪ Great diagnostic test performance, especially in conjunction with CA19-9; currently boasts highest reported AUROC values.

Abbreviations: ctDNA, circulating tumour DNA; CTCs, circulating tumour cells.

### 3.5. Multi-Cancer Early Detection Tests

Beyond explorations of PDAC in isolation, recent studies have also investigated multi-cancer early detection (MCED) tests that utilise liquid biopsy techniques. These assays seek to detect early-stage tumours for multiple cancer types, with a view for implementation as a population screening tool. Notable MCETs to date include *CancerSEEK* and *Galleri*.

*CancerSEEK* is a MCED test developed by Cohen et al. [92] able to detect eight common solid cancers using patient plasma, including pancreatic cancer. The test utilises a combination of ctDNA mutational analysis, involving multiplex PCR-based analysis of sixteen genes (61 amplicons), and measurement of eight circulating proteins via immunoassay. The study examined 1005 patients diagnosed with stage I-III non-metastatic tumours, including 93 patients with pancreatic cancer. Diagnostic sensitivity varied by cancer type from 69 to 98%, with an overall sensitivity for pancreatic cancer detection of 83.7% (and 99.5% specificity) [92]. Diagnostic performance improved with disease stage, with a reported test sensitivity of approximately 60% for stage I–II disease [93].

Conversely, *Galleri* is a MCED test developed by biotechnology company *GRAIL* based on data obtained from the Circulating Cell-free Genome Atlas (CCGA) study [94]. The test utilises a targeted ctDNA methylation assay combined with machine learning algorithms for population cancer screening [95]. The most recent substudy involved an analysis of 2823 patients across 27 types of cancer, including pancreatic cancer, spanning stages I-IV. Overall test sensitivity and specificity were 51.5% and 99.5%, respectively, with a higher-than-average sensitivity of 83.7% for pancreatic cancer. Like *CancerSEEK*, sensitivity generally increased with stage, reporting 61.9% sensitivity for stage I pancreatic cancer [94].

An additional two large, prospective, multi-centre, observational cohort studies are currently being conducted to clinically validate *Galleri* in intended-use populations [96]. *STRIVE* is a *GRAIL* study aiming to recruit 120,000 women undergoing screening mammography and is expected to be completed in 2025 [97], whilst *SUMMIT* is another *GRAIL* study intending to enrol 25,000 individuals at high risk for lung cancer and is expected to conclude in 2030 [98]. Furthermore, the *PATHFINDER* study was recently conducted to assess the feasibility of MCED testing for cancer screening by evaluating the impact of positive results on subsequent testing required for diagnostic resolution [96]. Schrag et al. examined 6662 participants prospectively, concluding overall that the study supported the viability of MCED screening; however, a positive predictive value (PPV) of just 38.0% was reported [99].

Therefore, whilst MCED testing may play a vital role in the ongoing development of crucial population screening tools for many cancers, including pancreatic cancer, it is evident further work is needed to achieve optimal clinical utility. Indeed, higher diagnostic sensitivities and improvements in PPV should be prioritised with future iterations of such technology.

## 4. Conclusions

The rising incidence, late detection, and lethality of PDAC presents an important health issue that necessitates the development of novel diagnostic, prognostic, and predictive biomarkers. Liquid biopsy techniques, including ctDNA, CTCs, circulating tumor exosomes, and proteomics, have emerged as potential solutions, offering the promise of transforming PDAC management and improving survival. Among these, proteomics stands out as a particularly promising avenue for further development, as new research highlights its superior diagnostic accuracy, as well as its potential prognostic value. However, further investigation is needed to validate existing findings, and comprehensively explore the proteome. Additionally, ongoing improvements to MCED testing performance are warranted for future development of effective population cancer screening tools.
